# A detailed Hapmap of the Sitosterolemia locus spanning 69 kb; differences between Caucasians and African-Americans

**DOI:** 10.1186/1471-2350-7-13

**Published:** 2006-02-28

**Authors:** Bhaswati Pandit, Gwang-Sook Ahn, Starr E Hazard, Derek Gordon, Shailendra B Patel

**Affiliations:** 1 Division of Endocrinology, Diabetes and Medical Genetics, Medical University of South Carolina, STR 541, 114 Doughty Street, Charleston, SC 29403, USA; 2 Biomolecular Computing Resource, Medical University of South Carolina, STR 541, 114 Doughty Street, Charleston, SC 29403, USA; 3 Lab of Statistical Genetics, Rockefeller University, 1230 York Avenue, NY 10021, USA; 4 Division of Endocrinology, Metabolism & Clinical Nutrition, Medical College of Wisconsin, 9200 West Wisconsin Avenue, E4950, Milwaukee, WI 53226, USA; 5 Department of Human Genetics, Mount Sinai School of Medicine, New York, NY 10029; 6 Department of Biology, College of Natural Science, Daejeon University, Daejeon 300-716, South Korea; 7 Department of Genetics, Rutgers, The State University of New Jersey, 145 Bevier Road, Room 128, Piscataway, NJ 08854-8009

## Abstract

**Background:**

Sitosterolemia is an autosomal recessive disorder that maps to the sitosterolemia locus, *STSL*, on human chromosome 2p21. Two genes, *ABCG5 *and *ABCG8*, comprise the *STSL *and mutations in either cause sitosterolemia. *ABCG5 *and *ABCG8 *are thought to have evolved by gene duplication event and are arranged in a head-to-head configuration. We report here a detailed characterization of the *STSL *in Caucasian and African-American cohorts.

**Methods:**

Caucasian and African-American DNA samples were genotypes for polymorphisms at the *STSL *locus and haplotype structures determined for this locus

**Results:**

In the Caucasian population, 13 variant single nucleotide polymorphisms (SNPs) were identified and resulting in 24 different haplotypes, compared to 11 SNPs in African-Americans resulting in 40 haplotypes. Three polymorphisms in *ABCG8 *were unique to the Caucasian population (E238L, INT10-50 and G575R), whereas one variant (A259V) was unique to the African-American population. Allele frequencies of SNPs varied also between these populations.

**Conclusion:**

We confirmed that despite their close proximity to each other, significantly more variations are present in *ABCG8 *compared to *ABCG5*. Pairwise D' values showed wide ranges of variation, indicating some of the SNPs were in strong linkage disequilibrium (LD) and some were not. LD was more prevalent in Caucasians than in African-Americans, as would be expected. These data will be useful in analyzing the proposed role of *STSL *in processes ranging from responsiveness to cholesterol-lowering drugs to selective sterol absorption.

## Background

Although our diets contain an equal amount of cholesterol and plant sterols, only 30–60% of cholesterol and less than 5% of total plant sterols are absorbed daily [1,2]. Additionally, of the small amounts of non-cholesterol sterols (primarily plant sterols) that are absorbed, these are preferentially excreted into bile by the liver, resulting in a very low level of whole-body retention [2]. In sitosterolemia, intestinal discrimination between cholesterol and non-cholesterol sterols and the ability of the liver to excrete normally all sterols (cholesterol and non-cholesterol sterols) are disrupted [3]. Thus, the defect in sitosterolemia defines the molecular mechanisms by which these processes take place.

We mapped the sitosterolemia disease to a single locus, *STSL*, to chromosome 2p21 in a region defined by the markers *D2S2294 *and *Afm210xe9 *[4-6]. This locus has now been shown to comprise of two highly homologous genes, *ABCG5 *and *ABCG8*, arranged in a head-to-head organization [7,8]. Two mutations in either both copies of *ABCG5 *or both copies of *ABCG8 *result in sitosterolemia [7-9]. To date, sitosterolemia has not been reported to be caused by a person harboring a mutation in one allele of *ABCG5 *and one allele of *ABCG8*. These gene are expressed in a tissue-specific manner (liver and intestine only) and they are thought to function as obligate heterodimers [10]. Genetic analyses of *STSL *showed that despite their close proximity, *ABCG8 *shows a much greater genetic variability than *ABCG5 *[8]. This disparate genetic evolution seems to be unique to humans, as the mouse and rat *STSL *loci show relatively equal extent of variations in *Abcg5 *and *Abcg8 *[11,12]. At present, the implications of the relatively more conservation in *ABCG5 *compared to *ABCG8 *is not known. This seems remarkable, since both genes are highly homologous to each other, with preserved exon-intron structures and are also highly conserved from Man to Fugu [12].

The human genome is arranged in an array of haplotype blocks (haploblocks), characterized by segments of high LD followed by regions of low LD [13-17]. Haploblocks may have arisen from recombination hotspots that are never divided during meiosis [18,19] or may be randomly distributed due to uniform but rare recombination [20].

In this paper, we report the detailed characterization of the SNPs present at the *STSL *in Caucasians drawn from our cohort of sitosterolemia families, as well as a group of African-American individuals who were normal and healthy. These data allows us to characterize this locus in detail and define some of these haploblocks. Preliminary reports have implicated *STSL *in physiological processes ranging from responsiveness to 'statin' drugs used to lower plasma cholesterol, as well as more complex processes such as the metabolic syndrome [21-29]. The data reported herein should allow for a more detailed and definitive testing of these hypotheses.

## Methods

### SNP analyses

All studies were performed after Institutional Review Board approval and with informed consent of the participants. Genomic DNA was isolated from blood obtained from Caucasian patients and their family members as previously described [8]. African-American DNA samples came from the ongoing Sea Islands Families Project/Project Sugar at the Medical University of South Carolina [30,31]. These individuals are part of the larger Gullah-speaking ethnic community who were born and reared in the coastal Sea Islands of Georgia, South Carolina and North Carolina, and whose parents were also reared on the Sea Islands. In the Project Sugar protocols, Type 2 diabetic probands are identified and then phenotypic data and DNA are obtained from the proband and the proband's family members. This database was screened for all individuals who were not diabetic and unrelated to each other to obtain a total of 46 unrelated individuals. Each exon and boundary intronic area of *ABCG5 *and *ABCG8 *was amplified by specific primers as previously described and SNPs detected by restriction enzyme digestion patterns [5,8] or by the primer extension method, using a capillary DNA analyzer (CEQ 8000, Beckman Coulter, Fullerton, CA). For the latter, amplified PCR products were digested with two units of Shrimp alkaline phosphatase (SAP, Roche Chemicals) and one unit of Exonuclease I (New England Biolabs, Ipswich, MA) at 37°C for one hour to remove unused primers and unincorporated nucleotides. The enzymes were inactivated by treating the samples at 75°C for 15 min. A primer extension reaction was set up using cleaned PCR products as template, the downstream primer adjacent to the SNP and fluorescent dideoxynucleotides. Multiplex extension reactions were carried out in some cases (details available on request). Samples were analyzed using a genetical analyzer, CEQ800 using the manufacturer's SNP separation method. Based upon the measured frequencies of each allele, observed genotypes were compared to expected genotypes for deviation from the Hardy-Weinberg principle. X^2 ^values were calculated by comparing the observed and expected genotype frequencies using the formula Σ(observed-expected value)^2^/Expected value. P value was obtained from the X^2 ^value table. The Age of mutation fixation was calculated as described by Guo and Xiong [32]. We selected 12 parents (24 chromosomes) carrying the commonest mutation, W361X, and where complete genotype information was available to compute recombination frequencies.

### Haplotype analyses

Genotyping data were used to estimate haplotypes using SNPHAP program [33], PHASE v2.1 [34] and haploblocks were constructed using HaploBlockFinder v6 [35]. Linkage disequilibrium measures [36], D' and Δ^2^, were estimated between pairs of diallelic loci using the value of Lewontin's D' [37] and measured using the GOLD program [38].

## Results

Our study consists of 32 parents (obligate carriers for mutations in either *ABCG5 *or *ABCG8*) of Caucasian origin from around the world [8]. Our African-American cohort consists of 46 unrelated individuals from the Sea Island community around South Carolina. Table 1 lists 23 SNPs (6 in *ABCG5 *and 17 in *ABCG8*) identified by extensive re-sequencing at the *STSL *locus by us, or those reported in the literature. The SNPS will be referred to as either by the amino acid they affect, or by their relative position in the gene. A formal identification for each of these SNPs is provided in Table 1. Only two loci in *ABCG5*, R50C and Q604E showed variations in our study population, the remaining 4 SNPs were invariant in all subjects and are not depicted in the haplotype analyses. For *ABCG8*, only 12 variable loci were identified, marked by an asterisk, Table 1, the remaining were non-variant in our study samples. All of the SNPs shown in Table 1 except M429V in ABCG8 were analyzed. M429V was reported only recently in a Japanese cohort [29], and was not included for analyses in this study. However, in limited analyses, we did not detect this in any of our previously sequenced exon 9 DNA traces either (data not shown), suggesting it may have a very low prevalence. Thus, only 14 SNPs (asterisked, Table 1) constitute the haplotypes shown and are ordered with *ABCG5*, followed by *ABCG8*.

**Table 1:** Polymorphisms reported at the *STSL *locus

Name	Position in the gene	Polymorphism	dbSNP cluster ID	Restriction enzyme site altered	Nucleotide Position
**ABCG5**					
P9P	Exon 1	C/T	rs49854016	BstN 1	22881725
R50C*	Exon 2	C/T	rs6756629	*-*	22881023
V523I	Exon 11	G/A	ss49854017	*-*	22863069
C600Y	Exon 13	G/A	ss49854018	*-*	22856345
Q604E*	Exon 13	G/C	**rs6720173**	*Sml I*	22856334
V622M	Exon 13	G/A	ss49854019	*-*	22856280
					
**ABCG8**					
5' UTR-41	5' UTR	C/T	ss49854020	*BstE II*	22882085
5' UTR-19*	5' UTR	T/G	**rs3806471**	*Tsp45 I*	22882107
P17P	Exon 1	G/C	ss49854021	*-*	22882176
D19H*	Exon 1	G/C	**rs11887534**	*-*	22882180
INT1-21*	Intron 1	C/A	ss4148209	*Mnl I*	22887558
INT1-7*	Intron 1	C/T	**ss4148210**	*BsmA 1*	22887572
C54Y*	Exon 2	G/A	**ss4148211**	*SexA I*	22887676
E238L*	Exon 6	G/A	ss49854010	*-*	22895692
A259V*	Exon 6	C/T	ss49854012	*Hae III*	22895756
Q340E	Exon 7	C/G	ss49854024	*-*	22915101
T400K*	Exon 8	C/A	**ss4148217**	*Mse I*	22915366
M429V	Exon 9	G/A		*-*	22916932
INT9-19	Intron 9	C/T	ss49854025	*-*	22917460
INT10-50*	Intron 10	C/T	**ss4148220**	*-*	22918168
A565A*	Exon 11	C/T	**ss4148221**	*-*	22918424
G575R*	Exon 11	G/C	rs49584011	*Hha I*	22918452
A632V*	Exon 13	C/T	rs6544718	*Sty I*	22920858

*Only these SNPs were found to be variant in the present study and the haplotypes (See Table 2 and 3) are ordered with these reported in sequence. The SNPs shown in bold (4^th ^column) are ones that also part of the HapMap dataset. Nucleotide numbering according to GenBank Sequence ID NT_022184.

The A259V polymorphism was present only in African-Americans. The C/T polymorphism at INT10-50 position, E238L and G575R in *ABCG8 *were variable only in the Caucasians. The haplotypes shown comprise all of the marked loci in both groups (Table 1) in order. Among the unrelated parents (Caucasians) all the SNPs, except R50C were in Hardy-Weinberg Equilibrium (p > 0.005, χ Square test). To completely characterize the haplotype structure, we estimated haplotype frequencies in each sample population using the multi-locus genotype data for each sample population. Estimates of haplotype frequencies are presented in Tables 2 and 3 for the Caucasians and African-Americans, respectively. These frequencies were estimated using the method described by Excoffier and Slatkin [33] as implemented in the SNPHAP program (Electronic Database Information).

**Table 2:** Estimated haplotype frequencies for Caucasians

Haplotype*	Est. Freq.	Cum. Freq.
11111111111111	0.229	0.229
11111111121111	0.224	0.453
11111121111111	0.083	0.536
11211121111111	0.063	0.599
11111121121111	0.042	0.641
21111111121111	0.031	0.672
21212221111112	0.031	0.703
11111121111112	0.031	0.734
11212221111111	0.031	0.766
21122221111111	0.016	0.781
21112111121112	0.016	0.797
21211221112111	0.016	0.813
11211121111211	0.016	0.828
11212211112111	0.016	0.844
21112221121111	0.016	0.859
11112122112112	0.016	0.875
12121111111112	0.016	0.891
11111121112112	0.016	0.906
22111111121111	0.016	0.922
11112121121111	0.016	0.938
11112221111112	0.016	0.953
21212221121212	0.016	0.969
11211111111122	0.016	0.984
11112111111111	0.016	1.000

*The left to right order of the haplotype reports on the asterisked SNPs shown top to bottom in Table 1.

**Table 3:** Estimated haplotype frequencies for African-Americans

Haplotype*	Est. Freq	Cum Freq
11111121111111	0.099	0.099
11111221121111	0.076	0.175
11111121111211	0.071	0.246
11111111111211	0.070	0.316
11111111121111	0.059	0.376
11111121121111	0.054	0.430
11111111111111	0.041	0.471
11111221111111	0.034	0.505
21111211121111	0.030	0.535
21112221111111	0.030	0.564
21111121121111	0.028	0.592
12121221111111	0.022	0.614
21212111111211	0.022	0.636
11211111111211	0.022	0.657
11121121221111	0.022	0.679
21111111121211	0.022	0.701
21111121111211	0.021	0.721
21111211111111	0.020	0.742
11112121121111	0.016	0.757
21111211111211	0.015	0.772
11112211111111	0.012	0.785
21111111121111	0.012	0.797
11211221211111	0.011	0.808
11211121111111	0.011	0.818
11221121111211	0.011	0.829
11211211111111	0.011	0.840
11211211111211	0.011	0.851
11112121221111	0.011	0.862
11121111111111	0.011	0.873
11111221211211	0.011	0.884
11112121111112	0.011	0.895
11221111111211	0.011	0.905
11111221121211	0.011	0.916
21121121111111	0.011	0.927
11111211221111	0.011	0.938
21112121221211	0.011	0.949
21112121211211	0.011	0.960
11211211211111	0.011	0.971
21111221111112	0.011	0.982
21122211111111	0.011	0.992

*The left to right order of the haplotype reports on the asterisked SNPs shown top to bottom in Table 1.

**Figure 1:**
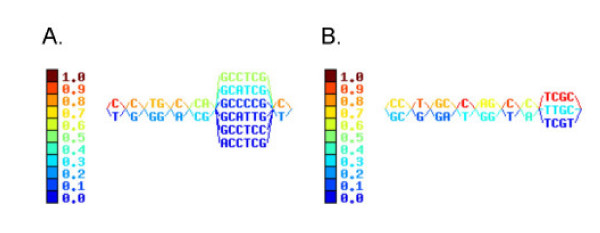
**Haploblock structure of *STSL *in Caucasians and in African-Americans**. Haploblocks were constructed as described in Methods. The color bars indicate the frequencies with which each of the haploblock sequences are found. Note the larger haploblock arrangements in Caucasians (panel A), compared to that observed in African-Americans (panel B).

The frequencies of the minor alleles varied from 0.02632 to 0.5 shown by different color code in Fig. 1. Twenty-four haplotypes were constructed from 64 chromosomes with the SNP signature CCTGCCGGCCTCGC haplotype as the most common among Caucasians, accounting for ~23% of the haplotypes (Table 2). For SNPs that affect amino acids, this translates to E604-R50-D19-C54-E238-A259-T400-A565-G575-A632. The next common Caucasian haplotype differs from this one in that there is a lysine at position 400 in ABCG8 (K400) and these two haplotypes account for ~45% of all haplotypes. There were many minor haplotypes whose contribution was very low (Table 2). The haplotypes were divided into seven blocks. The haplotype data is summarized in Table 2.

**Figure 2:**
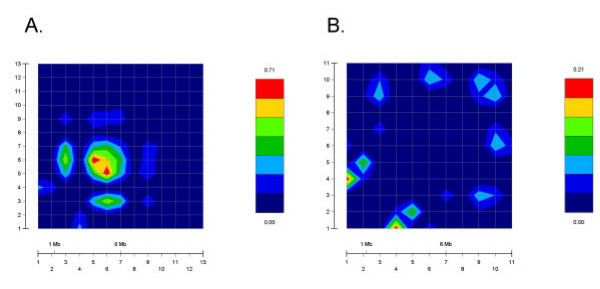
**GOLD plot of pair-wise Δ^2 ^values for SNPs in Caucasians and African-Americans**. As a pictorial depiction of LD at the *STSL*, GOLD plots for Caucasians (panel A) and African-Americans (panel B) were plotted. Note the differences in the color scales between the two panels shown on the right-hand side of each plot. Overall, a small area of LD was present in the Caucasian sample, as shown by the increased red intensity, almost all of this was accounted for an area involving intron 1. For African-Americans, very little LD was present (compare the GOLD plots with Table 4).

In the case of African-Americans, we identified four SNPs whose prevalence deviated significantly from Hardy-Weinberg equilibrium (5' UTR-19, D19H, A259V, A565A) all of which are located in ABCG8. Minor allele frequencies varied from 0.02174 to 0.38043. Haplotypes were divided into eight blocks as shown in Fig. 2. Additionally, a SNP that results in A259V in ABCG8 was detected in this cohort, but was absent in Caucasians, and a SNP that was variant in Caucasian (E238L in ABCG8) but was non-variant in African-Americans. The cumulative frequency shown (Table 3) does not sum to 1 in the African-American sample population. We deleted from the list of haplotypes those whose estimated frequencies were less than 1/(2 × 46) ~0.00107, since there are only 92 total haplotypes in all 46 individuals. Forty haplotypes were constructed, of which the signature CCTGCCGGCCTCGC was the major haplotype (~9.9%), translating to a coding haplotype of E604-R50-D19-C54-E238-A259-T400-A565-G575-A632, identical to the commonest Caucasian haplotype. However, the second commonest haplotype in Caucasians, with the K400 change was not detected at all in the African-Americans. Note the large numbers of haplotypes with the lower frequencies detected in the African-Americans, compared to the Caucasian samples.

Table 4 presents results of pair-wise linkage disequilibrium (LD) analysis for the fourteen SNPs at the *STSL *locus in these two populations (African-American and Caucasian). In this table, we present the pair of loci considered (columns labeled M1 and M2), the value of the Chi-square statistic (column labeled ChiSq) that tests whether *D' *(a measure of LD [37]) is non-zero, the p-value corresponding to the Chi-square statistic (column labeled Pval), and estimates of two measures of linkage disequilibrium, Δ^2 ^[36] and *D' *[37]. Both measures of linkage disequilibrium range between 0 and 1, 0 meaning no LD and 1 meaning complete disequilibrium. Although we computed LD values for (142)=91
 MathType@MTEF@5@5@+=feaafiart1ev1aaatCvAUfKttLearuWrP9MDH5MBPbIqV92AaeXatLxBI9gBaebbnrfifHhDYfgasaacH8akY=wiFfYdH8Gipec8Eeeu0xXdbba9frFj0=OqFfea0dXdd9vqai=hGuQ8kuc9pgc9s8qqaq=dirpe0xb9q8qiLsFr0=vr0=vr0dc8meaabaqaciaacaGaaeqabaqabeGadaaakeaadaqadaqaauaabeqaceaaaeaacqaIXaqmcqaI0aanaeaacqaIYaGmaaaacaGLOaGaayzkaaGaeyypa0JaeGyoaKJaeGymaedaaa@3410@ pairs of markers, we present results only for those pairs whose p-value for the Chi-square statistic is less than 0.10 in the interest of consolidation of results. Pair-wise LD was calculated using GOLD program (Fig. 2, regions red in color indicate high LD values). Caucasians (Fig. 2A) appear to have larger pair-wise Δ^2 ^for consecutive markers more frequently than do African-Americans (Fig. 2B). Of note, for the non-synonymous SNPs, R50C and D19H showed some LD in both populations, though the Ch-square statistic was only moderate (Table 4). Amongst Caucasians, the strongest LD was observed between the two intronic SNPs, INT1-12 and INT1-7, and to a lesser extent between INT1-7 and both 5'UTR-19 and Q604E (Table 4).

**Table 4:** Results of pair-wise LD analyses

Population	M1	M2	ChiSq	Pval	Δ^2^	*D'*
Caucasian	INT1-21	INT1-7	20.01	1E-05	0.545	0.866
	5'UTR-19	INT1-7	9.61	0.002	0.256	0.594
	Q604E	INT1-7	7.14	0.008	0.239	0.489
	T400K	A632V	6.13	0.013	0.125	1.000
	5'UTR-19	T400K	5.84	0.016	0.153	1.000
	Q604E	D19H	5.02	0.025	0.174	1.000
	INT1-7	T400K	4.94	0.026	0.111	1.000
	R50C	D19H	4.79	0.029	0.234	0.484
	INT1-21	T400K	4.45	0.035	0.153	1.000
	E238L	INT10-50	4.42	0.036	0.238	1.000
	INT1-7	C54Y	4.41	0.036	0.138	0.739
	5'UTR-19	C54Y	4.24	0.040	0.134	0.619
	T400K	INT10-50	3.92	0.048	0.040	1.000
	5'UTR-19	A565A	3.86	0.049	0.127	1.000
	Q604E	INT1-21	3.66	0.056	0.128	0.420
	INT10-50	A632V	3.29	0.070	0.132	0.641
	5'UTR-19	INT1-21	2.86	0.091	0.071	0.267
	C54Y	T400K	2.74	0.098	0.082	0.433
						
African-American	5'UTR-19	T400K	11.01	9E-04	0.080	1.000
	INT1-7	A565A	8.09	0.004	0.085	0.587
	R50C	D19H	6.96	0.008	0.205	1.000
	T400K	A565A	6.56	0.010	0.088	0.557
	Q604E	INT1-21	5.82	0.016	0.119	0.505
	5'UTR-19	A565A	5.10	0.024	0.059	0.460
	C54Y	A565A	3.93	0.047	0.053	0.270
	5'UTR-19	C54Y	3.49	0.062	0.047	0.481
	R50C	INT1-7	3.05	0.081	0.044	1.000
	INT1-7	A632V	3.05	0.081	0.044	1.000
	Q604E	D19H	3.01	0.083	0.038	1.000

M1 = 1^st ^marker in pair of SNPs, M2 = 2^nd ^marker in pair of SNPs, ChiSq = Value of chi-square test of association, Pval = Two-sided P-value corresponding to chi-square value in ChiSq column assuming 1 degree of freedom.

Δ^2 ^= Estimated value of Delta-squared measure of LD.

D' = Estimated value of Lewontin's [37] measure of LD.

**Figure 3:**
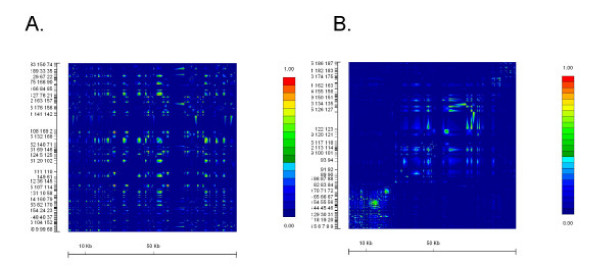
**GOLD plot of pair-wise Δ^2 ^values for SNPs in CEPH and Yoruba Africans genotyped by the HapMap Consortium**. We analyzed the SNP genotypes spanning the *STSL *locus for the CEPH (panel A) and Yoruba (panel B) samples available at . The HapMap SNP dataset are much more dense. Note that the Yoruba samples show very little LD compared to the Caucasian samples. Additionally, the Caucasian samples also show that the *STSL *locus does not have any large segments of LD.

**Figure 4:**
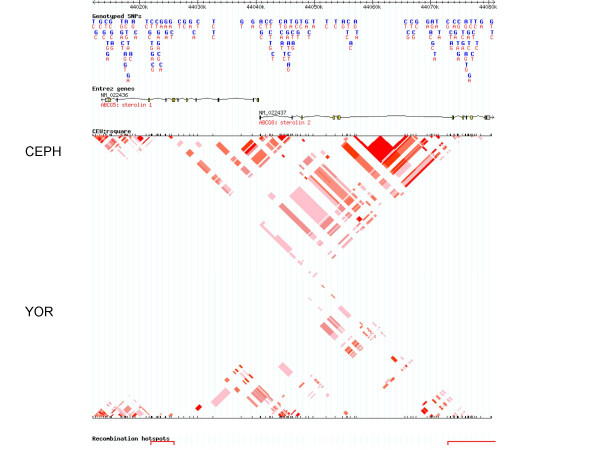
**Haploview analyses of CEPH and Yoruba Africans genotyped by the HapMap Consortium**. Using the data and analyses available at the website, , we selected the genotypes for the CEPH and Yoruba populations. The CEPH LD plot, using Haploview is shown above the Yoruba plot (inverted). As can be seen, for both, there is little evidence of significant LD for the SNP makers used. Additionally, two hotspots for recombination were identified (depicted by the red bars at the bottom of the picture), located at the ends of the *STSL *locus and involve the terminal exons of both ABCG5 and ABCG8 (which contain the transmembrane domains).

With the publication of the HapMap data during the preparation and submission of this manuscript [39], we were able to compare our data with that of the HapMap data (available at [40]). We compared data for SNPs typed on chromosome 2, between positions 44,012,000 to 44,081,000, containing the *STSL *locus. The GOLD plots for Caucasian samples (CEPH family data) and African samples (Yoruba samples from Nigeria) are shown in Fig. 3. The HapMap data for this region is much denser. Additionally, there are some significant differences between SNPs used in our study and those reported by the HapMap Consortium. Firstly, of the 14 SNPs we found were variant in our entire cohort, only 8 of these were also genotyped by the HapMap Consortium, highlighted in Table 1. Secondly, of the 6 that are unique to our genotyping, 5 of these are cSNPs and are all non-synonymous changes, and the 6^th^, INT1-21, was one where we detected significant LD for Caucasian samples (See Fig. 2A). Of the remaining 9 SNPs we genotyped and found no variants (Table 1), with the exception of M429V, which we did not genotype, the HapMap Consortium also do not report any genotyping data. It is not clear to us whether this is because they also failed to show these were variant, or because these were not tested. Nevertheless, given the richness of the HapMap dataset, we present the Haploview analyses (which allows for a better pictorial representation than GOLD for large data sets) for these two populations (Fig. 4), which are essentially the screen-shots available at their website [40], with some image cropping for presentation. Note that there are 2 hot-spots of recombination that can be identified and these are located at the ends of both ABCG5 and ABCG8 (Fig. 4). Despite differences highlighted between the HapMap Data and ours, the overall conclusions are similar; in both analyses, the samples originating in peoples from Africa show the least amount of linkage disequilibrium, the greatest variability and smaller haploblocks (data not shown), compared to Caucasian samples. The differences between our samples may also be significant. Our Caucasian samples are drawn from families with sitosterolemia and come from many different parts of the World. Our African-American samples, while maintaining a much closer genetic tie to Africa, are drawn from peoples from a variety of Africans originating from West Africa, not just the Yoruba, in Nigeria [30].

Age of mutation was calculated considering W361X as the most common disease causing mutation. Table 5 summarizes the data linking the estimated age of mutation with the alleles. Of the non-synonymous cSNPs, T400K of ABCG8 was found to be oldest polymorphism that arose about 2387 generations ago (~47,000 y). This SNP was also closely spaced to W361X mutation. The youngest cSNPS was estimated to be ~180 y old (C54Y). The only cSNP we could estimate by this methodology in ABCG5 was Q604E (~4,000 y old). We could not estimate the ages in some of the SNPs due to insufficient information to allow us to estimate recombination frequencies (indicated by NA, Table 5).

**Table 5:** Estimation of age of polymorphism fixation.

SNP	Allele	Number of Disease Chromosomes*	Number of Healthy Chromosomes*	Frequency (disease chromosome)	Frequency (healthy chromosome)	Recombination Fraction	Age Estimate (generations)
R50C	C	12	12	1	1	NA	
Q604E	G	2	1	0.167	0.083	0.058833	17.7
5'UTR-19	T	11	10	0.917	0.833	0.033059	9.1
D19H	G	12	12	1	1	NA	NA
INT1-21	C	12	7	1	0.583	0.005	0
INT1-7	C	11	11	0.917	0.917	NA	
C54Y	A	9	5	0.75	0.417	0.02749	8.8
E238L	G	12	12	1	1	NA	
T400K	A	10	3	0.833	0.25	0.0002	2387
INT10-50	T	12	12	1	1	NA	
A565A	C	12	12	1	1	NA	
G575R	G	12	12	1	1	NA	
A632V	C	11	10	0.917	0.833	0.005692	52.9

*Out of a total of 12 disease and 12 normal chromosomes, see Methods

## Discussion

There are several reports correlating defects in a polygenic disease with single nucleotide changes in the coding or regulatory regions. SNPs also offer a substantial advantage in linkage disequilibrium-based studies of disease gene mapping [41], pharmacogenetics [42] and human evolution [43]. Studies of African, Asian and European Caucasian populations have shown that both a dense marker set, as well as larger sample size will be needed for a stable fine-scale depiction of haploblocks [44,45]. Variations in *APOD *gene were associated with an increased risk of early onset of Alzheimer's disease in a group of Finns [46]. Responses to pharmacotherapy also vary from person to person and in part can be accounted for by genetic variations and haplotype structures [47]. Thus characterization of SNPs, as well as the haploblock structures will be useful in defining the roles of genes in health and disease. We have characterized SNPs present at the *STSL *locus in Caucasian families with sitosterolemia and in a group of normal healthy African-Americans. This locus is important as its disruption leads to the human disorder, sitosterolemia [3]. More importantly, this locus comprises of two genes, *ABCG5 *and *ABCG8*, which are critical in handling of dietary sterols and for biliary sterol excretion [48]. Thus they are important in whole body sterol balance and have been implicated in cardiovascular health. A number of studies have been implicated this locus in disease (or physiological) processes ranging from lipoprotein kinetics [22], cholesterol absorption [27,29], obesity [27] to response to drug therapy [26].

When performing power and sample size calculations for disease or QTL genetic association, a critical parameter is some measure of linkage disequilibrium between the trait and marker locus [49-52]. Because the trait locus is unobserved, this parameter is usually unknown. A surrogate measure is some average marker-marker linkage disequilibrium measure [53]. Our work determining marker-marker linkage disequilibrium for SNPs in the *ABCG5/ABCG8 *gene cluster will enable researchers to perform more realistic power and sample size calculations for genetic association studies involving the *ABCG5/ABCG8 *cluster. Prior to placing these studies in context of the data reported herein, there are some important points that need highlighting about our study. While this manuscript was in submission, the HapMap data were reported [39]. This latter dataset is not only more dense, it examined 4 different populations. The Chinese and Japanese samples show significantly more LD over this area, with much larger haploblock structures than the Yoruba and the CEPH populations (data not show, but available at [40]). These data are in keeping with our analyses of the high degree of homozygosity for markers spanning the STSL locus in families with sitosterolemia originating from Japan or China [5]. However, comparison of the SNPs we genotyped to those in the HapMap dataset showed that a significant number of cSNPs we genotyped were not analyzed in the HapMap dataset (see Table 1). Additionally, one cSNP, M429V, which was reported to be relatively more frequent in the Japanese population [29], was also absent from the HapMap dataset analyzing the Chinese Han and the Tokyo Japanese DNA samples. Thus our analyses presented herein add to this body of knowledge.

We noted a number of differences between the Caucasian and African-American populations. Some of these are expected. For example, the African-American population sampled contains many more variations and haplotypes. Additionally, the haploblock structures were smaller and the extent of linkage disequilibrium between markers was lower, in keeping with the Out-of-Africa theory for the origins of humans. This was true for both our dataset as well as analyses of the HapMap dataset. Some exceptions are notable. SNPs in intron 1 of *ABCG8 *show some linkage to a common non-synonymous SNP, Q604E, in *ABCG5*, but present in exon 13 (almost 20 kb apart). It is not clear if the intronic variations have a regulatory effect on transcription, but these data draw attention to this possibility. The transcriptional regulation of *STSL *remains poorly characterized, with few definitive studies to indicate which regulatory transcriptional factors, as well as nucleotide sequences are involved.

Four SNPs, 5' UTR-19, D19H, A259V, and A565A, in *ABCG8 *were not in Hardy-Weinberg equilibrium for the African-Americans. One explanation is sampling error. Since the unrelated African-American samples were gleaned from a sample collected for the presence of diabetes and family members ascertained, it is possible that, despite genealogical screens to remove related samples etc., some non-randomness error has skewed the data. This error may be compounded by the small sample size. Another explanation is that there may be a selection process that has led to this. We favor the first explanation, although this issue will only be resolved with analyses of a much larger sample. Secondly, we confirm that despite the relative proximity of *ABCG5 *to *ABCG8*, there was significantly less variation observed for *ABCG5 *and would suggest some selection or difference in mutational rates and fixation between *ABCG5 *and *ABCG8*. Since *ABCG5 *and *ABCG8 *are proposed to function as obligate heterodimers [54], and complete mutations in either gene seems to result in an identical phenotype [8], these genetic findings posit an enigma. It is not clear what selective pressures may be responsible for this, if any. In rodents, almost equal variations are noted in *Abcg5 *and *Abcg8 *[11,12]. It will be of some interest to see if this difference in *ABCG5/ABCG8 *variability is present in populations related to Man, such as Chimpanzees and other greater apes. We note that in the HapMap dataset, there are many more SNPs reported for ABCG5. However, all of these are exclusively located in the non-coding regions and, to date, there are more cSNPs in ABCG8 than there are in ABCG5. A number of association studies reporting linkage of certain SNPs at the *STSL *locus to a number of seemingly unconnected phenotypes, ranging from response to a cholesterol-lowering drug, to insulin sensitivity and lipoprotein kinetics in obese subjects have been reported. Unfortunately, these do not intuitively allow for a selective advantage, positive or negative, that can explain the differences in the variability between *ABCG5 *and *ABCG8*.

Compared to other markers, SNPs have a lower mutation rate and are valuable for estimating age of mutations. SNPs in *ABCG5 *appear to be newly created compared to those in ABCG8. Additionally, in this study, we could not replicate the identification of other polymorphic variants in *ABCG5*, including some we have reported previously [8]. These are P9P, V532I and V622M. This may reflect the rarity of these SNPs and our small sample size. If so, to investigate the role of these SNPs in biology may require a much larger sample size. Additionally, these SNPs may be race-related. For example, the M429V SNP was reported in the Japanese samples and seems to play a role in cholesterol absorption [29]. However, we were not able to detect this SNP in either the Caucasian or the African-American samples. Additionally this SNP was also not reported in the Japanese and Chinese cohort in the HapMap dataset. Thus, this SNP may represent another race-specific polymorphism.

## Conclusion

We report a detailed characterization of the *STSL *locus, present data that show regions of LD at this locus and provide data that should allow for more accurate Power calculations for studies examining the role of this locus in human biology. Our dataset has uniquely analyzed SNPS not reported in by the SNP consortium and therefore add to this knowledge base.

## Abbreviations

ABC; ATP binding cassette, SNP; single nucleotide polymorphism. *STSL*; sitosterolemia locus.

## Competing interests

The author(s) declare that they have no competing interests.

## Authors' contributions

BP, G-SA, SEH, DG and SBP generated data and analyzed them. BP and SBP wrote the manuscript.

## Pre-publication history

The pre-publication history for this paper can be accessed here:


